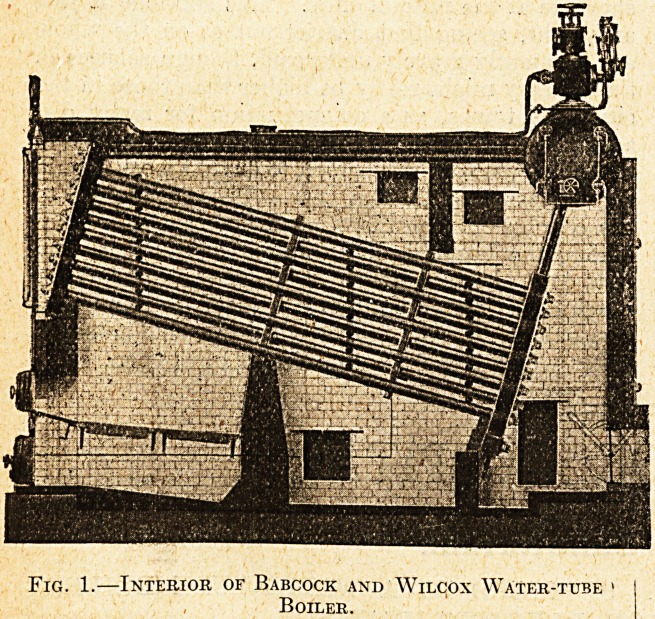# Pinewood Sanatorium

**Published:** 1917-01-13

**Authors:** 


### THE HEATING OF HOSPITALS.

### XI. Pinewood Sanatorium.

### LOW-PRESSURE STEAM RADIATORS, SUPPLIED BY A WATER-TUBE BOILER WITH A SMALL QUANTITY OF ELECTRIC HEATING.

Pinewood is a consumptive sanatorium situated in a clearing in the pine woods in the neighbourhood of Reading. It consists of a number of separate buildings, and has sixty-four separate patients' rooms opening out of corridors, besides the administration block, billiard and games rooms, gymnasium, and the quarters for the staff.

It is one of the few hospital buildings in which the water-tube boiler is employed, and its use in this case is due to the fact that the boiler and all heating-appliances are shut down at 9.30 p.m. until the morning. The patients' rooms are heated by low-pressure steam radiators, one in each room, and there are sixty-one other steam radiators distributed about the corridors, bathrooms, administration block, etc. The billiard-room, the games-room, and the gymnasium are heated by large steam coils.

The steam for the whole of the radiators, coils, etc., is furnished by two Babcock and Wilcox water-tube boilers, each capable of evaporating 200 gallons of water per hour, to a pressure of 120 lb. per square inch; they are usually worked at 80 lb. per square inch. The steam is delivered to the buildings by means of pipes, ranging from 4 inches diameter at the boiler-house down to 1¼ inch at the furthest building; the size of the pipes decreases as the distance from the boiler increases, and the quantity of steam to be carried decreases. The pressure of the steam is reduced to a little over 5 lb. per square inch in the boiler-house by a reducing valve in the usual way; and in the radiators and steam coils is at 5 lb. per square inch. The pipes are insulated with non-conducting composition, and are all laid in trenches in the ground, except one which is insulated in a special manner. This pipe is laid on the surface of the ground inside a second pipe; the outer pipe is protected by a covering of non-conducting composition. It will be remembered that still, dry air is a non-conductor of heat, and the engineer's idea in this case is that the layer of air and the composition coating the outer pipe give better insulation than the composition on the pipes laid in the ground, while the pipe can be very easily got at for repairs. A special form of joint is made in this pipe, consisting of sleeves on the two pipes; that on the outer one is covered with composition. In case of a leak at a joint the outer sleeve can be slipped along the pipe, and the joint of the inner pipe easily got at for repair. It is the engineer's experience at Pinewood that with the pipes in the trenches a leak has to be very pronounced before it receives attention, on account of the difficulty of getting at the pipes. No special attempt is made to control the waste of steam between the boiler-house and the radiators.

A second set of pipes, to which all the radiators are connected, carries the water formed by the condensation of the steam back to the hot-well, situated in the boiler-house, from which it is pumped into the boiler. The steam pipes are inclined slightly

Fig. 1.—Interior of Babcock and Wilcox Water-tube Boiler.

downwards as they recede from the boiler, and steam traps are fitted at the end of each steam pipe; so that all the water of condensation formed in the steam pipes is carried forward to the steam traps, which deliver to the system of return-pipes, leading to the hot-well. The steam pipes are led to the neighbourhood of each radiator, and connection is made between the steam pipe and one side of the radiator; the admission of steam to the individual radiators being controlled by the usual wheel valve. In practice it is usual to put the valve wide open when heating is commenced in the morning, and when any room becomes too hot, to shut it right off, turning it on again when the temperature has fallen.

### The Hot-water Supply.

Hot water is supplied to each building during the time the boiler is working by a calorifier similar to those described in previous articles. The calorifiers take their steam from the same pipes that supply the radiators, at a pressure of 5 lb. per square inch. The steam spaces of the calorifiers are connected to steam traps, which deliver the water of condensation to the pipe system described above, leading to the hot-well. After the boiler is shut down hot water is supplied from an insulated tank on the roof, holding 200 gallons of water, which is filled during the day. The insulation of the tank prevents the water cooling, in the same manner as the insulation of the pipe prevents the condensation of the steam. The engineer estimates that the insulated tank, the calorifiers, and the pipes together hold about 250 gallons of water, whose temperature ranges from 140° Fahr. upwards, so that there is a plentiful supply of hot water available right through the night.

### The Water-tube Boiler *v.* The Tank Boiler.

The Americans call Lancashire and Cornish boilers tank boilers: they are virtually large cylindrical tanks containing water and steam, where the water space is pierced by the tubes carrying the furnaces and the hot gases formed by combustion. They are particularly suitable for hospital work because of their simplicity and reliability. The large volume of hot water and steam they carry enables them to respond to very heavy calls, and there is no difficulty in keeping them under steam continuously for months together. As hospitals nearly always carry at least one spare boiler, one of those in service can always be shut down at any convenient time, and allowed to cool; and it can then be quite easily inspected, and any necessary repairs carried out. The boiler inspectors sent out by the boiler insurance companies usually get right inside the boiler and make careful inspection of every part. Workmen also can get inside to do repairs. It has one drawback for certain classes of work; it requires six hours to get up steam from the cold state, as time has to be allowed to heat up the large mass of metal of which the boiler consists, so that the joints between the plates may not be strained by unequal expansion. In the case of hospitals this is usually not a source of great trouble, as the boilers are generally wanted to be under steam continuously; it is only under conditions such as rule at Pinewood that it is of importance. The water-tube boiler will raise steam from the cold state in an hour, or even less, and hence its advantage for Pinewood. Fig. 1 is a drawing showing the structural arrangement of a Babcock and Wilcox water-tube boiler. The water circulates in all water-tube boilers through a number of tubes, the hot gases formed by the combustion of the coal passing all over the outside of the tubes. There is always a cylinder, much smaller than those used in tank boilers, carried above the tubes; sometimes there are two or three such cylinders. The steam, as it is formed, is delivered to one of them, and the feed water is usually delivered to another, from which it flows to the tubes that are at the lowest temperature, and thence through the other tubes, a portion of the tubes usually containing only steam. The furnaces (there are usually two) are

(*Continued on page* vi.)

## Figures and Tables

**Fig. 1. f1:**